# Estimation of temporal covariances in pathogen dynamics using Bayesian multivariate autoregressive models

**DOI:** 10.1371/journal.pcbi.1007492

**Published:** 2019-12-13

**Authors:** Colette Mair, Sema Nickbakhsh, Richard Reeve, Jim McMenamin, Arlene Reynolds, Rory N. Gunson, Pablo R. Murcia, Louise Matthews

**Affiliations:** 1 MRC-University of Glasgow Centre for Virus Research, Institute of Infection, Immunity and Inflammation, College of Medical, Veterinary and Life Sciences, University of Glasgow, Glasgow, United Kingdom; 2 School of Mathematics and Statistics, College of Science and Engineering, University of Glasgow, Glasgow, United Kingdom; 3 Boyd Orr Centre for Population and Ecosystem Health, Institute of Biodiversity, Animal Health and Comparative Medicine, College of Medical, Veterinary and Life Sciences, University of Glasgow, Glasgow, United Kingdom; 4 Health Protection Scotland, NHS National Services Scotland, Glasgow, United Kingdom; 5 West of Scotland Specialist Virology Centre, NHS Greater Glasgow and Clyde, Glasgow, United Kingdom; University of California, Los Angeles, UNITED STATES

## Abstract

It is well recognised that animal and plant pathogens form complex ecological communities of interacting organisms within their hosts, and there is growing interest in the health implications of such pathogen interactions. Although community ecology approaches have been used to identify pathogen interactions at the within-host scale, methodologies enabling robust identification of interactions from population-scale data such as that available from health authorities are lacking. To address this gap, we developed a statistical framework that jointly identifies interactions between multiple viruses from contemporaneous non-stationary infection time series. Our conceptual approach is derived from a Bayesian multivariate disease mapping framework. Importantly, our approach captures within- and between-year dependencies in infection risk while controlling for confounding factors such as seasonality, demographics and infection frequencies, allowing genuine pathogen interactions to be distinguished from simple correlations. We validated our framework using a broad range of synthetic data. We then applied it to diagnostic data available for five respiratory viruses co-circulating in a major urban population between 2005 and 2013: adenovirus, human coronavirus, human metapneumovirus, influenza B virus and respiratory syncytial virus. We found positive and negative covariances indicative of epidemiological interactions among specific virus pairs. This statistical framework enables a community ecology perspective to be applied to infectious disease epidemiology with important utility for public health planning and preparedness.

## Author summary

Disease-causing microorganisms, including viruses, bacteria, protozoa and fungi, form complex communities within animals and plants. These microorganisms can coexist harmoniously or even beneficially, or they may competitively interact for host resources. Well-studied examples include interactions between viruses and bacteria in the respiratory tract. Whilst ecological studies have revealed that some pathogens do interact within their hosts, identifying interactions from available population scale data from health authorities is challenging. This is exacerbated by a lack of large-scale data describing the infection patterns of multiple pathogens within single populations over long time frames. Furthermore, methods for evaluating whether infection frequencies of different pathogens fluctuate together or not over time cannot readily account for alternative explanations. For example, human pathogens may have related seasonal patterns depending on the age groups they infect and the weather conditions they survive in, and not because they are interacting. We developed a robust statistical framework to identify pathogen-pathogen interactions from population scale diagnostic data. This framework serves as a crucial step in identifying such important interactions and will guide new studies to elucidate their underpinning mechanisms. This will have important consequences for public health preparedness and the design of effective disease control interventions.

This is a *PLOS Computational Biology* Methods paper.

## Introduction

Animals and plants are exposed to a wide range of pathogenic organisms that co-circulate in time and space. When multiple pathogens infect the same tissue, they form diverse communities, effectively sharing an ecological niche that provides the opportunity for interspecific interactions [1–3]. It is known that pathogen interactions may alter the within-host dynamics of infection with consequences for the population transmission of some common infections. Interactions among microorganisms include the promoting or inhibiting effects of gut microbiota on invading pathogenic bacteria in the gastrointestinal tract [4]; the enhanced carriage of pneumococcal bacteria following influenza infection in the respiratory tract [5]; the rise in human monkeypox after eradication of smallpox [6]; and immune-driven enhancement of Zika virus infection following Dengue virus exposure [7]. The complex ecology of pathogen communities therefore has potentially important implications for the epidemiology and control of infectious diseases.

Pathogens that act non-independently and their health implications is an actively growing and important area of research [8]. Pathogen interactions can be cooperative or competitive and can occur within a host or in a population where pathogens co-circulate [9]. While some evidence of population-level interactions between pathogens exists, statistical support for the occurrence of pathogen-pathogen interactions from multiple non-stationary time series independent of prior biological or ecological knowledge is lacking. This is due in part to a paucity of appropriate long-term time series data that describe infection frequencies for multiple pathogens simultaneously, allowing such interactions to be identified, but also due to statistical techniques that are limited in their ability to handle such complex datasets [10, 11].

Various statistical methods are available to analyse health-related time series data. Statistical methods for handling non-stationary time series data include multiple regression and generalised additive models, which are able to capture non-linear trends and explanatory factors such as seasonality and climate as well as other confounders and typically model a univariate health outcome as opposed to a multivariate distribution of several non-independent outcomes [12–18]. Consequently, they do not necessarily focus on estimating pathogen-pathogen interactions. More specialist techniques that focus on decomposition of the time series include singular spectrum analysis and wavelet analysis. Singular spectrum analysis has been used to model interactions between a pathogen and an environmental factor [19], whilst wavelet decomposition has been used to infer pathogen-pathogen interactions [20] and virus-virus interactions [21]. These techniques only capture pairwise relationships between time series (for example pathogen-pathogen or pathogen-environment) although in principal singular spectrum analysis can be extended to multiple time series [22]. Moreover these methods do not account for or adjust the data for potential confounders. Another recent approach is to use mechanistic stochastic models to estimate time varying parameters (e.g. a transmission rate) and then employ wavelet analysis to compare with potential weather or climatic drivers [11], but again in a pairwise manner.

Alternative approaches that focus specifically on identifying interactions include confirmatory analyses that fit observed time series data from two pathogens to models containing hypothesised interactions [9, 23]. Extending to multiple pathogens increases the complexity of this approach [9]. Confirmatory analyses rely on prior biological and ecological knowledge in order to hypothesize an appropriate model with interpretable parameters. Specifically, the ‘true’ interaction needs to be modelled and therefore such analyses cannot capture unexpected or unknown interactions [10].

In contrast, exploratory approaches such as Granger-Causality and Transfer Entropy can provide robust statistical evidence for unknown interactions from multiple time series whilst accounting for confounding variables [24], and have been used to detect virus-virus interactions [10]. However, they rely on stationarity of the times series, and non-stationarity can generate spurious results [25]. This limits the applicability of this approach to many epidemiological time series since seasonality and long term trends (and therefore non-stationarity) is a long-recognised attribute of many infectious diseases [26].

A framework that can infer unknown interactions from multiple pathogens incorporating non-stationary time series data whilst adjusting for confounding factors will advance this important research area [10]. Here, we construct just such a robust framework, which is able to identify pathogen-pathogen interactions from multiple non-stationary time series at the population scale independent of prior biological or ecological knowledge.

The conceptual framework for our new approach derives from Bayesian disease mapping models—a class of regression model that has received much attention in recent years for the analysis of spatial distributions of incidence data routinely collected by public health bodies [27, 28]. These models are typically applied to incidence data to estimate spatial patterns of disease risk over a geographical region—with several models proposed to capture spatial autocorrelations [19] using conditional autoregressive priors [29, 30]. While some extensions to disease mapping models have been made to include temporal patterns [29, 31] and space-time interactions [32, 33], most disease mapping applications focus on spatial structures [34] with temporal dependencies in disease incidence often being overlooked [35, 36].

Modelling multiple pathogens simultaneously allows assessment of related patterns and non-independence of infection risk. Multivariate forms of disease mapping models provide a suitable framework for estimating temporal dependencies between pathogens as they naturally incorporate a between-disease (or pathogen) covariance matrix [37]. In this paper, we construct a framework for time series data analysis that allows the estimation of covariances among temporal disease datasets. Because the approach accounts for confounding variables and sources of non-stationarity such as seasonally varying infection risk, the resulting statistical framework now enables the joint estimation of pathogen dependencies on the temporal dimension whilst, crucially, distinguishing genuine pathogen-pathogen interactions from simple correlations.

To validate our method we conducted extensive simulation studies using synthetic data. We then applied the method to diagnostic data on five respiratory viruses (adenovirus [AdV], coronavirus [Cov], human metapneumovirus [MPV], influenza B virus [IBV] and respiratory syncytial virus [RSV]) from the patient population of a major urban UK population (Glasgow, United Kingdom) over a period of nine years. We chose this particular group of pathogens because i) respiratory viruses are obligate intracellular pathogens that have a strong predilection for the cells of the respiratory tract (i.e. they share the same ecological niche); ii) contemporary diagnostic tests based on multiplex real-time PCR (qPCR) technology allow the simultaneous detection of multiple respiratory viruses from the same patient; and iii) multiplex qPCR was routinely used to diagnose respiratory viruses in the patient population of Glasgow during the 2005-2013 period.

## Modelling approach

**Fig 1:**
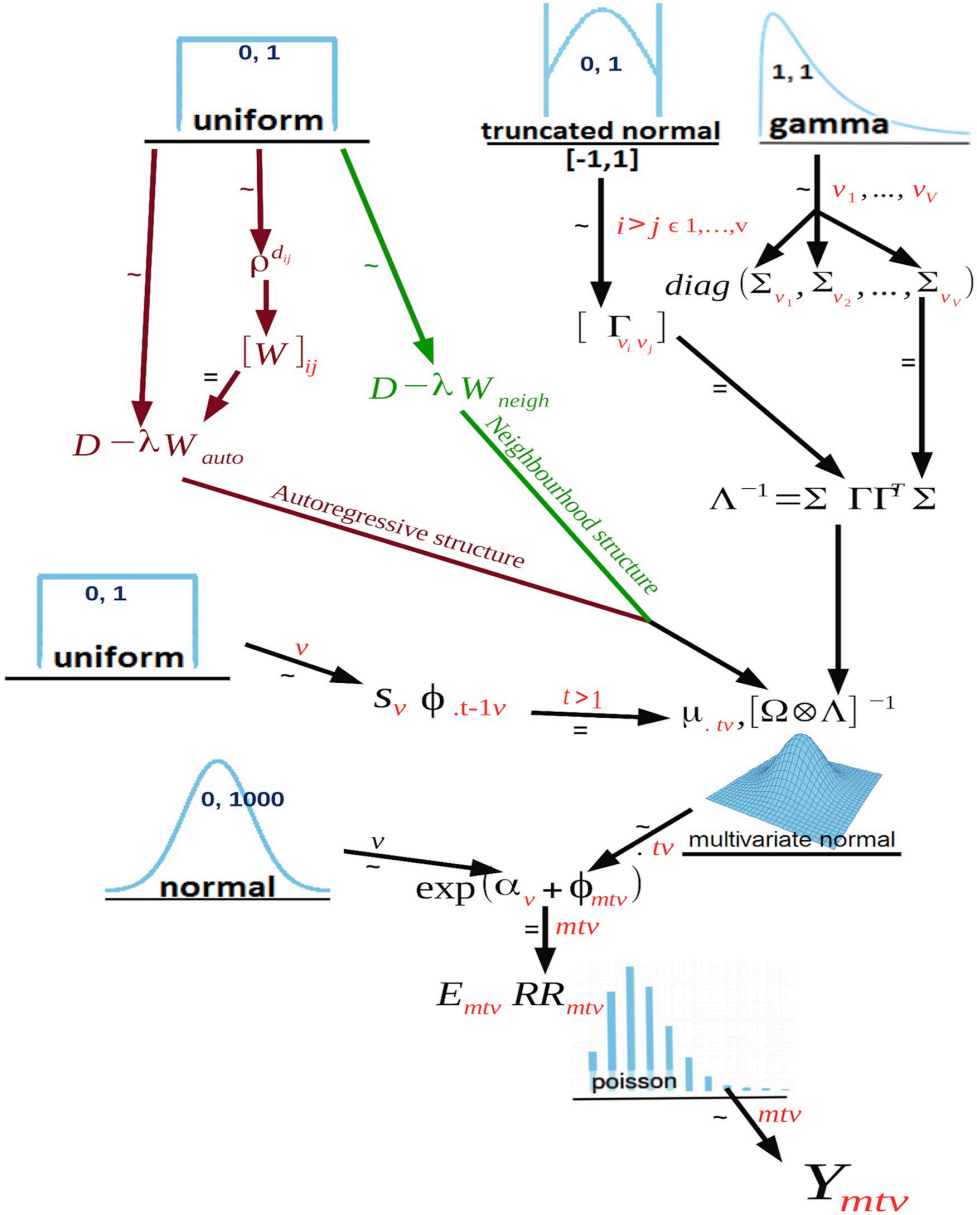
Model used to estimate pairwise relative risk covariances. The diagram should be read from the bottom (starting with *Y*_*mtv*_) to the top. All prior choices have been fully specified. Numbers indicate hyperparameter choices, for instance, mean and variance in the normal distribution, lower and upper bound in the uniform distributions and shape and rate in the gamma distribution. Numbers in red indicate all relevant subscripts month *m* = 1, …, 12, year *t* = 1, …, 9 and virus *v* = 1, …, 5. Green arrows correspond to the neighbourhood structure and maroon arrows correspond to the autoregressive structure.

The framework presented infers unknown pathogen interactions adjusted for confounding factors such as seasonality, demographics and testing frequencies using time series data from multiple contemporaneous pathogens (Fig 1).

We used *Y*_*mtv*_ to denote the observed count of pathogen *v* during the *m*th month of year *t* conditional on expected count *E*_*mtv*_ and relative risk *RR*_*mtv*_

$$\begin{array}{c} \begin{array}{cc} Y_{mtv}|E_{mtv},RR_{mtv} & ∼\text{Poisson}(E_{mtv}RR_{mtv})\\ \log(RR_{mtv}) & =\alpha_{v}+\phi_{mtv} \end{array} \end{array}$$

with *α*_*v*_ an intercept term specific to virus *v* and *ϕ*_.*t*._ = {*ϕ*_.*t*1_, …, *ϕ*_.*tV*_} a vector of random effects modelled conditionally through a MCAR prior

$$\begin{array}{ccc} \phi_{.t.}|\phi_{.t-1.} & ∼ & \text{MVN}(s_{v}\phi_{.t-1.},{\left[\Omega ⊗\Lambda \right]}^{-1}). \end{array}$$

Estimating expected counts enables us to adjust for potential and established confounding factors. For instance, the virus diagnostic data allowed us to use age, sex, whether the patient had attended a general practice or hospital (as a proxy for infection severity), month of year and testing frequencies. Therefore, expected counts explained a proportion of the variation in the observed counts and we attributed the remaining unexplained variation to temporal autocorrelation, virus-virus interactions and residual random variation.

The temporal autocorrelation is handled by adapting the approach from MCAR (Multivariate Conditional Autoregressive) models, designed to model spatially autocorrelated data based on neighbourhood relationships. Here, the parameterisation of a MCAR model captured both the seasonal trends of each pathogen via precision matrix Ω and non-independence between pathogens via Λ. Temporal effects *ϕ*_.*t*._ captured long term temporal tends with smoothing parameters *s*_1_, …, *s*_*V*_. Dependency structures between neighbouring months accounted for seasonality in pathogen infection frequencies. Two such structures were considered, namely the neighbourhood structure (Fig 1 green arrows), where all neighbouring months are equally correlated to the month in question, and the autoregressive structure (Fig 1 maroon arrows), where there is a power law weighting the correlation between related months and the month in question.

This method focuses primarily on the estimation of pathogen covariance matrix Λ^−1^. By formally testing which off-diagonal entries of ${\hat{\Lambda }}^{-1}$ are significantly different from zero, we can explicitly provide statistical support for pathogen interactions.

## Results

### Simulation study

In order to validate the proposed method, we performed an extensive simulation study using synthetic virus diagnostic data with a wide range of time series structures and estimated the power and type 1 error rate (i.e. rejection of the true null hypothesis) of this method for a range of correlations between viruses.

Individual level data of age, sex and general practice versus hospital attendance (a proxy for infection severity) were simulated to reflect the real virus diagnostic data, and the probabilities of infection for each virus within each month were estimated. For a full data description, we refer readers to Nickbakhsh et al [38]. Within each year, the number of samples tested for each virus per month ranged from 20 to 200 to reflect variable testing frequencies. Expected counts were calculated through standardised infection probabilities and testing frequencies.

**Fig 2:**
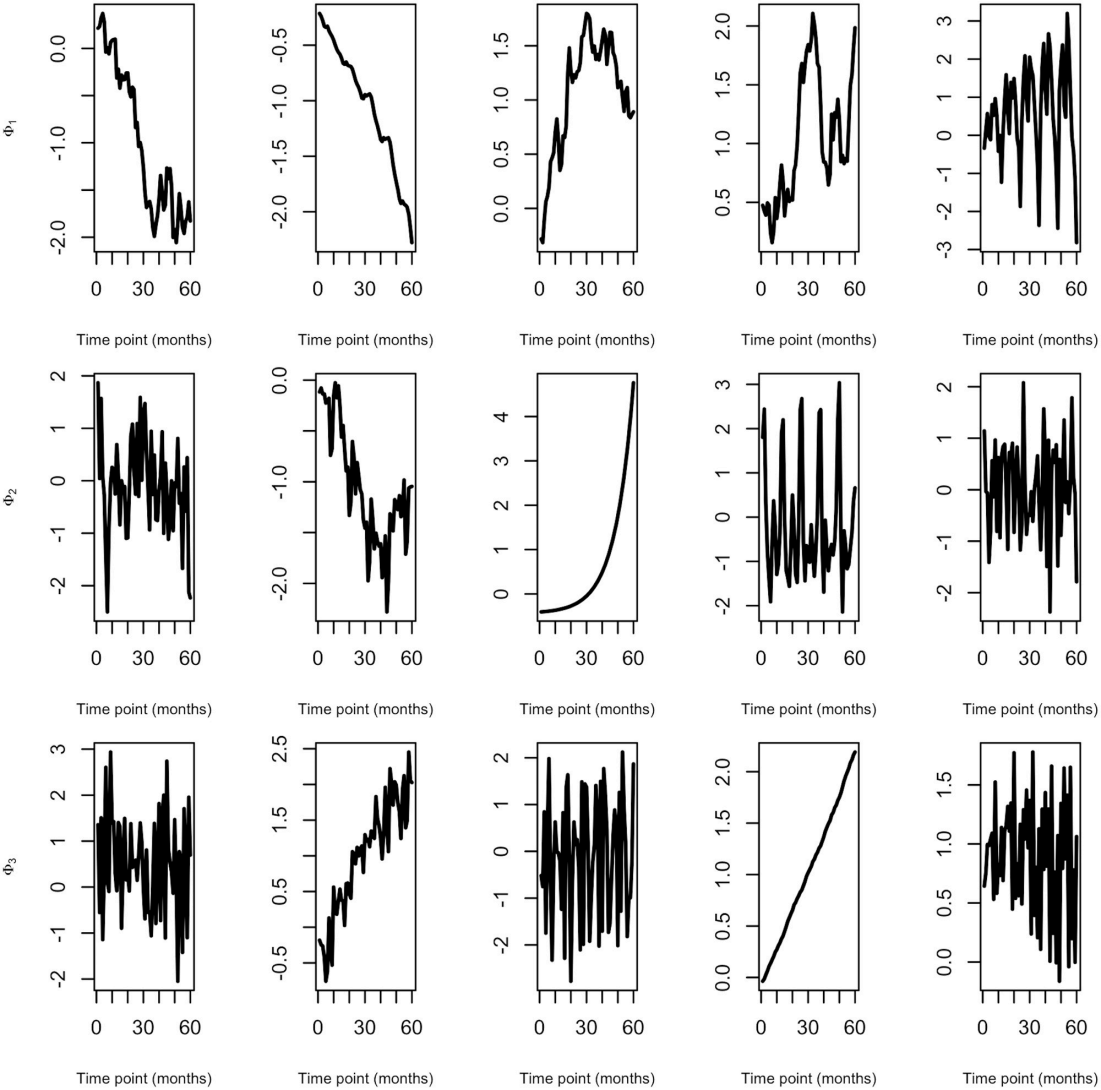
Examples of simulated temporal effects (*ϕ*_..*v*_) for three viruses. Illustrations of seasonal autoregressive integrated moving average time series data simulated under parameter settings used in simulation study.

Generation of the matrix Ω depended on the choice of correlation structure (either neighbourhood or autoregressive). Relative risks were calculated from the virus specific intercept term *α*_*v*_, simulated uniformly, and monthly effect terms *ϕ*_..*v*_. Monthly effect sizes were simulated without constraining the nature of time series data in order to illustrate the flexibility of this framework (Fig 2).

**Fig 3:**
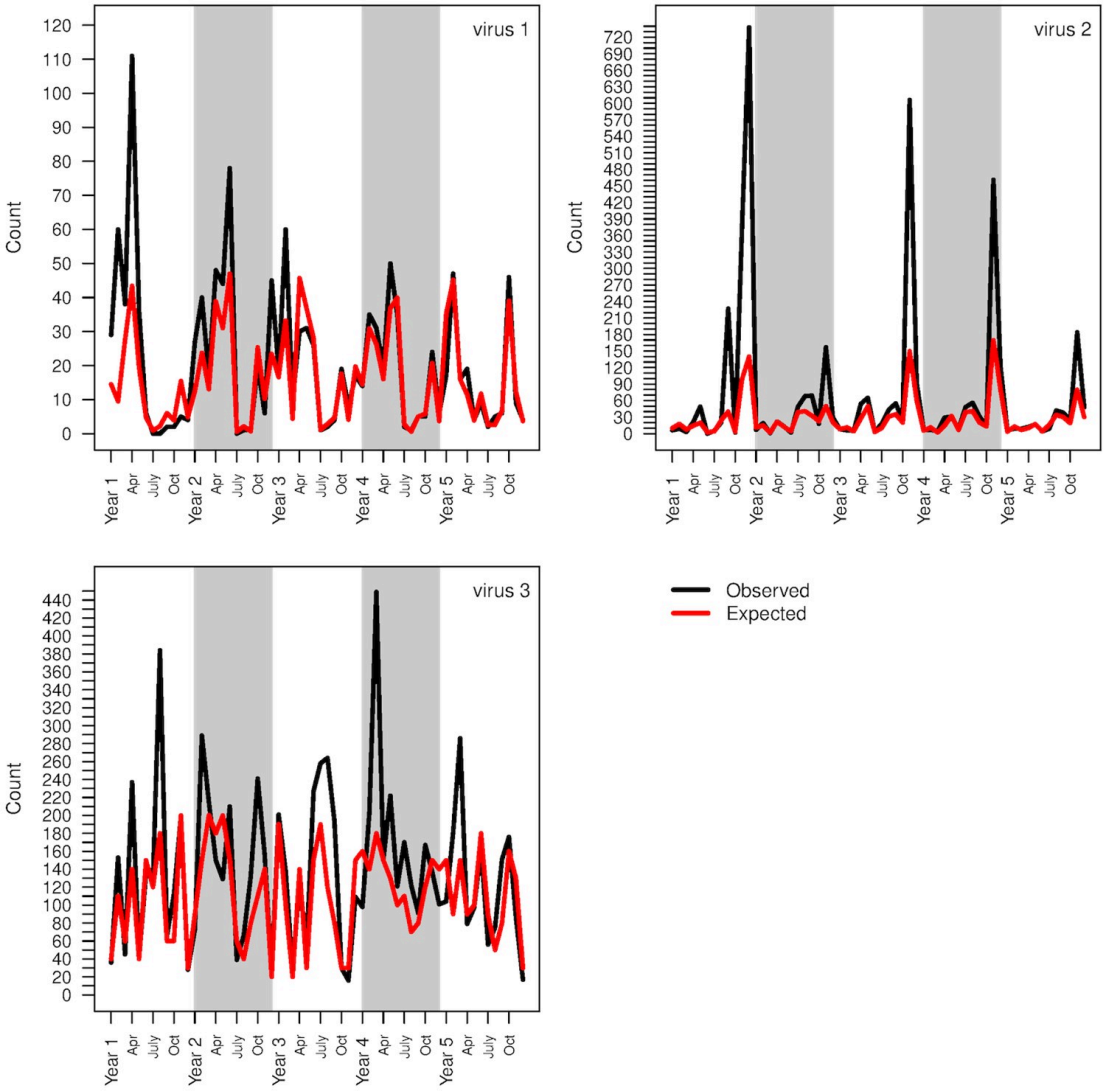
Example of simulated observed and expected counts. An example of observed and expected counts simulated from three viruses using the method described in the simulation study section.

An example of data simulated under the neighbourhood structure is present in Fig 3. A full description of the simulation setup and parameter choices is given in the material and methods.

Since our approach incorporated two structures that captured monthly autocorrelations (the neighbourhood structure (N) and autoregressive structure (A) either adjusting for multiple comparisons (post-mcc) or not (pre-mcc)), four possible combinations of simulation (Sim) and estimation (Est) are reported (Table 1). A range of correlations between two viruses were considered from weakly related viruses (correlation = 0.2) to a moderately strong correlation (correlation = 0.5) based on data simulated from three viruses over five years with two viruses correlated and the remaining virus independent.

**Table 1:** Simulation structure.

Simulating model (Sim)	Estimation model (Est)
N	N
A	A
A	N
N	A

Combinations used to simulate data and estimate model parameters using either the neighbourhood (N) or autoregressive (A) structures.

#### Power and type 1 error control

**Fig 4:**
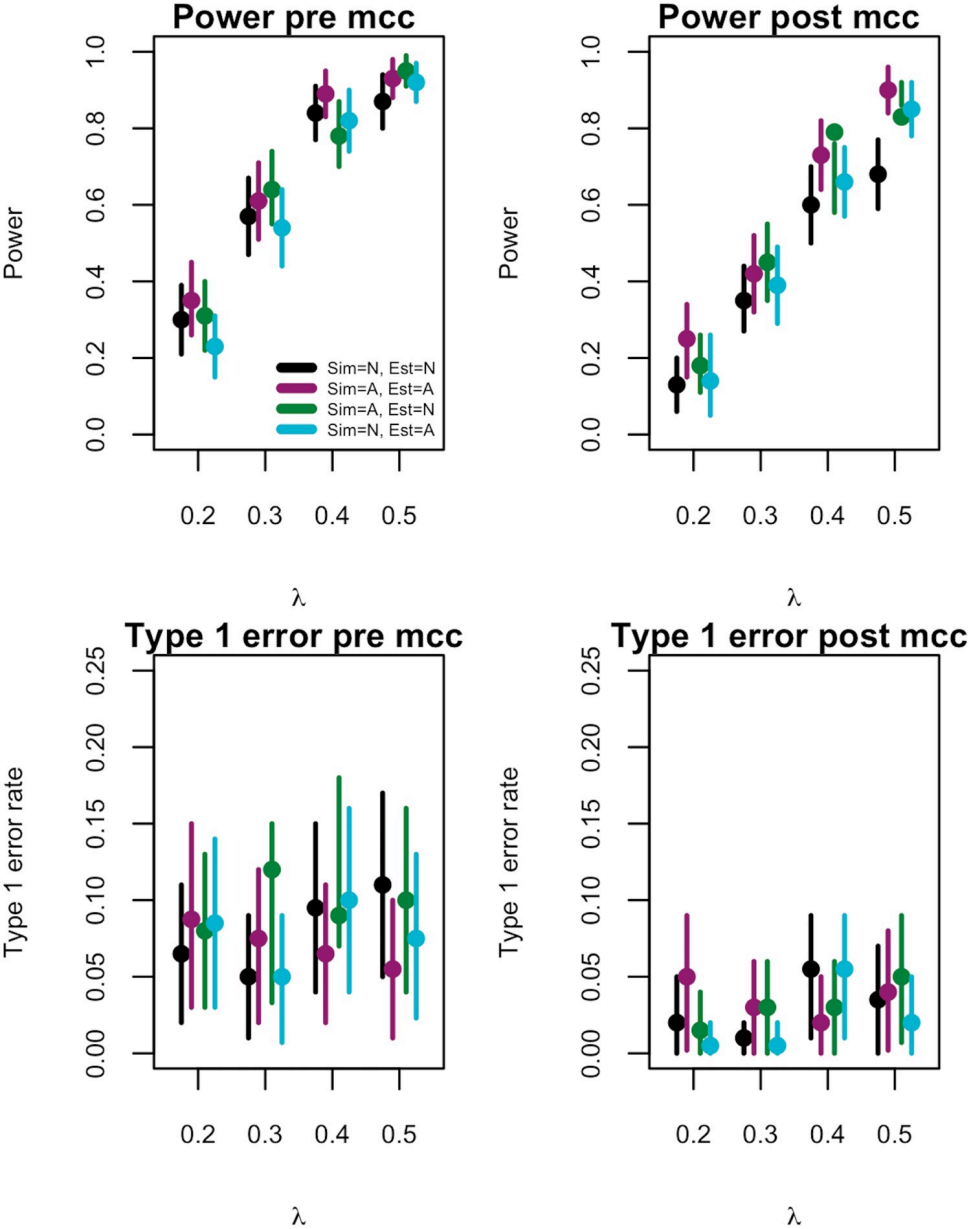
Power and type 1 error rate. Estimated power (top) and type 1 error (bottom) based on analysis of synthetic data for three viruses. Data were simulated (Sim) under one of two structures, neighbourhood (N) and autoregressive (A) and parameters estimated (Est) under one of the two structures. Results shown for no multiple comparison correction (pre-mcc), left, and with a multiple comparison correction (post-mcc), right.

Without correcting for multiple comparisons (pre-mcc) the power of detecting a moderately strong correlation of 0.5 was greater than 0.8 under each of the four scenarios (Fig 4, power pre-mcc). As expected, as the strength of the relationship between viruses increased, the power also increased. On the other hand, this test was unable to adequately control the type 1 error rate at a 5% significance level without correcting for multiple comparisons (Fig 4, Type 1 error pre-mcc). Therefore, as the number of related viruses increased, we were more likely to infer false relationships between viruses.

After correcting for multiple comparisons, the power of the test ranged from around 0.2 in the case of weakly correlated viruses (Fig 4, power post mcc). As expected, power decreased after correcting for multiple comparisons. We were able to precisely and accurately estimate, and generally found better control of, the type 1 error rate after correcting for multiple comparisons. However, we found no significant difference in the type 1 error rate pre and post multiple comparison correction (Fig 4, type 1 error pre and post mcc).

Overall, we found the autoregressive model to be more powerful in inferring correlations between viruses (Fig 4, power post mcc, purple line) with the least amount of success inferring correlations with the neighbourhood model (Fig 4, power post mcc black, lines). For instance the autoregressive model had an estimated power of 0.9 when λ = 0.5 whereas the neighbourhood model had an estimated power of 0.68.

### Virus diagnostic data

**Fig 5:**
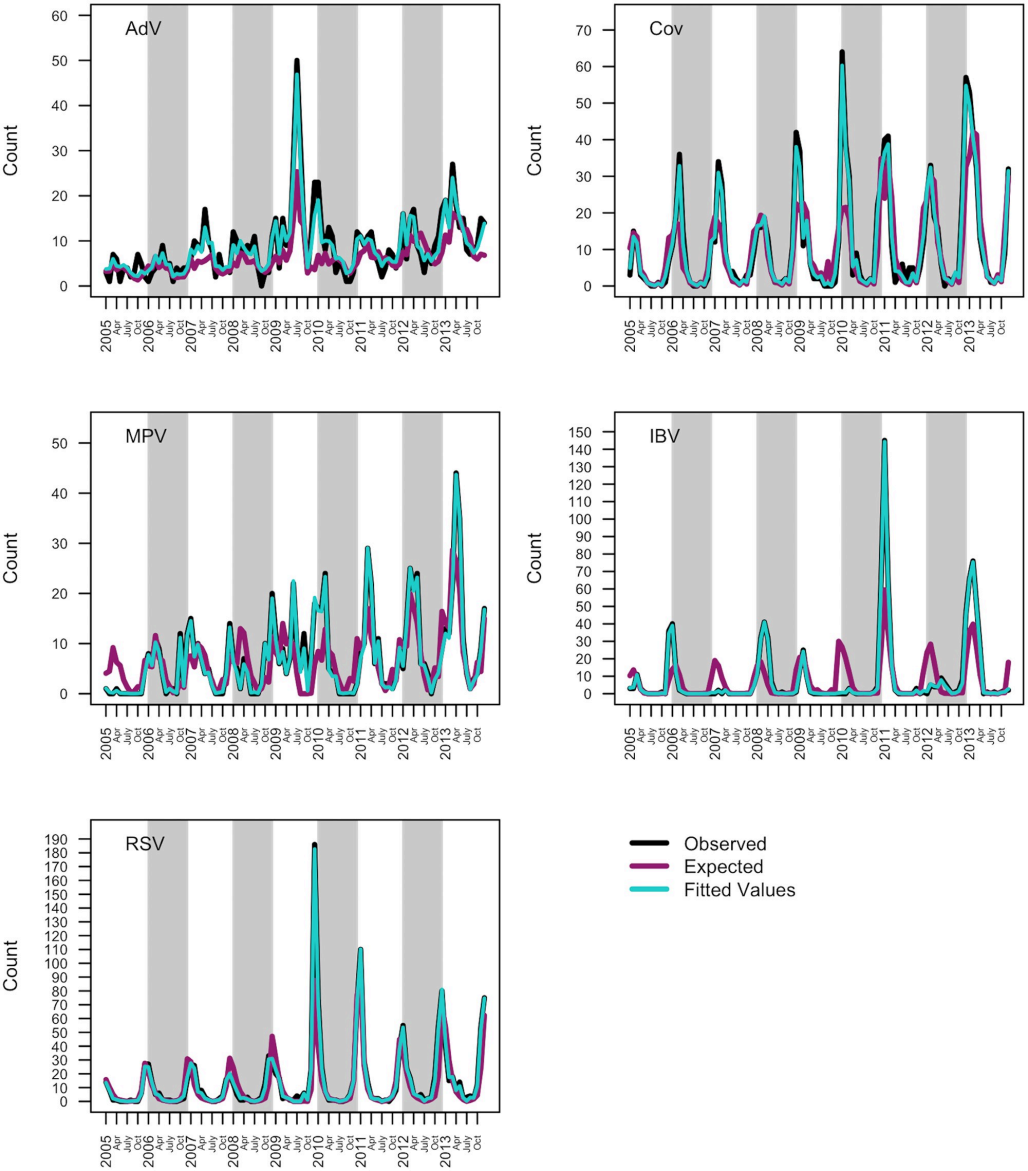
Observed, expected and fitted counts of AdV, hCov, hMPV, IBV and RSV. Observed (black), expected (purple) and fitted (light blue) counts of the five groups of respiratory viruses between January 2005 and December 2013. A full description of the estimated expected counts is given in the expected count section. Fitted values are based on autoregressive model.

From the 28,647 patient episodes, defined as aggregated samples taken from each patient over a 30-day window, 4,759 were positive to at least one virus group and detection was most common in children aged between 1 and 5 years. Detection of any virus in a given episode was most common in December and least common in August. We observed differing patterns between the five viruses (Fig 5, black lines). IBV, RSV and CoV were more prevalent in winter months (November, December and January), AdV was generally less common with a slight increase in prevalence in spring months (April, May and June) and MPV shifts from winter peaks (January and February) to spring peaks (March, April and May) after 2010. IBV was the only virus not to display a regular seasonal pattern. This virus peaked in winter during 2005/2006, 2007/2008, 2008/2009, 2010/2011 and 2012/2013 but failed to peak during the winter periods 2006/2007, 2009/2010 and 2011/2012.

#### Estimated infection expected counts

Expected counts were estimated for each virus and shown in Fig 5 (purple lines). The expected number of infections of AdV infection remained relatively high between 2005 to 2010 but decreased during the summer and autumn months of 2011, 2012 and 2013. We found an increased expected number of IBV infection during the autumn and winter periods of 2005/2006, 2010/2011 and 2012/2013. During the second half of 2009, we found a heightened risk of RSV and MPV infections. More generally, the risk of RSV infection peaked during late summer through to autumn from 2008 onwards whereas the risk of MPV infection shifted from winter, between 2005 and 2008, to summer, from 2011 onwards.

### Virus-virus interactions

For comparison, we first fitted a null model that assumed all five viruses to be independent by setting Λ^−1^ = *I*_5_ (the identity matrix of dimension 5 × 5). Under the neighbourhood structure, we found that allowing dependencies between viruses (Λ^−1^ ≠ *I*_5_) provided a better fit to the data (DIC = 2795.6 versus DIC = 3583.8 for the null model). However, the autoregressive structure with Λ^−1^ ≠ *I*_5_ minimised DIC (DIC = 2686.4).

Comparing observed values to fitted values under the autoregressive model fit (Fig 5, black and light blue lines respectively for each virus) to informally check model fit, we were able to accurately and precisely estimate observed counts of each virus across the nine year time period. Correlations between observed and fitted values ranged from 0.96 (p-value < 0.001) for AdV and 0.9997 (p-value < 0.001) for IBV (S2 Appendix).

More precisely, our model captured winter peaks in CoV, winter and spring peaks in MPV and irregularities in AdV and IBV validating the model fit to these data.

Under the neighbourhood structure, we found a positive covariance between RSV/MPV and negative covariances between IBV/MPV, CoV/MPV and AdV/IBV (Table 2, *W*_*neigh*_). Under the autoregressive structure, we found a positive covariance between RSV/MPV and a negative covariance between IBV/AdV (Table 2, *W*_*auto*_), with adjusted p-values for the covariances between IBV/MPV and CoV/MPV of 0.075 and 0.073 respectively.

**Table 2:** Estimated covariances between AdV, Cov, MPV, IBV and RSV.

		*W*_*neigh*_	*W*_*auto*_
Adv	CoV	(-0.27, 0.45)	(-0.31, 0.41)
	MPV	(-0.35, 0.22)	(-0.35, 0.20)
	IBV	**(-0.67, -0.16)**	**(-0.68, -0.15)**
	RSV	(-0.37, 0.29)	(-0.32, 0.41)
CoV	MPV	**(-0.66, -0.11)**	(-0.66, -0.08)
	IBV	(-0.23, 0.45)	(-0.18, 0.43)
	RSV	(-0.28, 0.32)	(-0.32, 0.29)
MPV	IBV	**(-0.66 -0.13)**	(-0.64, -0.07)
	RSV	**(0.32, 0.71)**	**(0.18, 0.67)**
IBV	RSV	(-0.51, 0.05)	(-0.54, 0.04)

Posterior density interval estimates of covariances between AdV, CoV, MPV, IBV and RSV. Covariances different from zero after multiple comparison correction are highlighted in bold.

Our analysis showed robust statistical evidence of a facilitative form of interaction between RSV and MPV and a competitive form of interaction between IBV and AdV.

## Discussion

Humans, animals and plants are exposed to a plethora of co-circulating pathogens, creating frequent opportunity for interactions between them. There is a growing interest in the health implications of interacting pathogens that has led to the development of new research in healthcare [8]. However, robust statistical methods to identify and quantify interactions among multiple pathogens have been lacking.

Traditional regression-based approaches can handle confounding variables but do not necessarily infer non-independencies between multiple response variables [12–18]. Time series specific methods (e.g. wavelets or spectral analysis) are powerful but do not handle confounding variables, are limited to pairwise comparison, and may also make assumptions of non-stationarity (e.g. Granger-Causality) [19–22, 24, 25]. Fitting epidemiological models which contain interactions to the data is also possible, but becomes very complex when multiple pathogens are present.

This paper addresses the need for a more widely applicable statistical framework that can jointly infer unknown interactions among pathogens for which multiple contemporaneous time series are available. The framework accounts for non-stationarity, confounding variables such as seasonality and patient demographics and requires no prior knowledge or specification of the underlying biological or ecological mechanisms.

We presented a conceptual framework derived from Bayesian multivariate disease mapping methods that provides a powerful statistical tool for inferring pathogen-pathogen interactions from diagnostic and/or surveillance time series data. Whilst standard multivariate disease mapping frameworks investigate the joint spatial distribution of multiple diseases coinfecting a population simultaneously, our method instead analyses the joint temporal distribution of multiple infections. Because multivariate disease mapping naturally incorporates a between-disease covariance matrix, these methods conveniently lend themselves to the inference of temporal signatures of pathogen-pathogen interactions when adapted to analyse temporal dependencies. Importantly, because our method accounts for confounding variables as well as the autocorrelation structure, the method distinguishes genuine pathogen-pathogen interactions from simple correlations.

By applying our framework to extensive diagnostic data accrued over a nine-year period from a well-defined patient population, our analysis provides evidence of epidemiological interactions among respiratory viruses. Acute respiratory infections are a significant cause of illness and mortality and are primarily attributed to a group of viruses that occupy a shared ecological niche in the respiratory tract. Although observational data [39–42] and univariate response regression models [41, 43–45] indicate the potential for interactions among these common pathogens, limited evidence exists of their impact on epidemiological infection dynamics. Under the autoregressive structure, which provided a better fit to these data, our analysis provides robust evidence of a positive covariance between RSV and MPV and a negative covariance between IBV and AdV. This provides a basis for future work to explore the public health implications of these relationships.

We anticipate that this framework will aid in the epidemiological understanding of linked pathogen dynamics. The knowledge that specific pathogen-pathogen interactions exist and of their form (positive or negative) provides an important first step towards improving disease forecasting models. Such models could be adapted for multi-pathogen systems by incorporating pathogen-pathogen interactions through reduced or enhanced transmissibility of secondary/co-infecting pathogens. Ultimately, improved understanding of the impact of coinfections on health outcomes will improve the public health utility of such models by enabling estimation of disease burden and pressures on different sections of the healthcare system, for instance the numbers of hospital beds needed at different times of the year.

In summary, we have developed a new and robust method of inferring interactions from multiple pathogen time series. Applying this approach to time series data of pathogens that co-circulate in a given population allows quantification of interactions that will lead to a better understanding of the joint epidemiological dynamics of diseases. These inferences, in combination with laboratory experiments to further elucidate the underlying mechanisms, will enhance the understanding of linked pathogen dynamics, inform the forecasting of disease incidence and improve public health preparedness. In addition, they will result in better ways to evaluate the impact of public health interventions, thus aiding the design of better measures to control infectious diseases.

## Materials and methods

### Respiratory virus infection time series data

Our dataset derives from routinely collected clinical samples tested for respiratory viruses by the West of Scotland Specialist Virology Center (WoSSVC) for Greater Glasgow and Clyde Health Board between January 2005 and December 2013. Each sample was tested by multiplex real-time RT-PCR and test results (virus positive or negative) were available for five groups of respiratory viruses: adenovirus [AdV]; coronavirus [CoV]; human metapneumovirus [MPV]; influenza B virus [IBV]; and respiratory syncytial virus [RSV] [46]. Sampling date, patient age, patient gender and sample origin (hospital or general practice submission that we used as a proxy for infection severity) were recorded. Multiple samples from the same patient received within a 30-day period were aggregated into a single episode of respiratory illness resulting in 28,647 patient episodes. A patient was considered virus-positive during an episode if at least one clinical sample was positive during the 30-day window. Ethical approval was not required here since samples were collected as part of routine diagnostic work. Information from NHS Scotland [47–49] informed participating patients of the use of their data. We refer the reader to Nickbakhsh et al. [38] for a full description of these data.

Whilst data are available at the individual level, we are predominantly interested in estimating correlations in temporal patterns between the five viruses at the population level. Therefore, for each virus, data were aggregated into monthly infection counts across the time frame of this study.

Relative risks identify time points where observed counts are higher or lower than expected, with expected counts accounting for expected seasonality and risk factors associated with respiratory infection [38]. We note that this differs from the conventional measure which compares exposed and unexposed groups. We used the relative risk to measure the excess risk of viral infection that cannot be explained by seasonality or patient demographics. By inferring dependencies between viral species in terms of excess risks, we can directly infer viral interactions.

### Multivariate spatio-temporal model

Conditional autoregressive models are extensively used in the analysis of spatial data to model the relative risk of a virus or more generally a disease [50, 51]. The class of Bayesian model typically used in this context is given by

$$\begin{array}{c} \begin{array}{cc} Y_{i}|E_{i},RR_{i} & ∼\text{Poisson}(E_{i}RR_{i})\\ \log(RR_{i}) & =\alpha +\phi_{i} \end{array} \end{array}$$

where *Y*_*i*_, *E*_*i*_ and *RR*_*i*_ are the observed count, expected count, derived from available patient demographic data (refer to expected counts section), and relative risk for some index *i* (for example, location or time interval) [30] and *ϕ* = {*ϕ*_1_, …, *ϕ*_*I*_} spatial random effects modelled jointly through a conditional autoregressive (CAR) distribution [52]

$$\begin{array}{ccc} \phi & ∼ & \text{MVN}(0,{\left(\tau \left(D-\lambda W\right)\right)}^{-1}). \end{array}$$

Matrix *W* is a proximity matrix, λ a smoothing parameter, *τ* a measure of precision and *D* a diagonal matrix such that *D*_*i*_ = ∑_*i*′_ *W*_*ii*′_.

Extending this model to multiple viruses, or more generally multiple pathogens, then

$$\begin{array}{c} \begin{array}{cc} Y_{iv}|E_{iv},RR_{iv} & ∼\text{Poisson}(E_{iv}RR_{iv})\\ \log\;\left(RR_{iv}\right) & =\alpha_{v}+\phi_{iv} \end{array} \end{array}$$

where *Y*_*iv*_, *E*_*iv*_ and *RR*_*iv*_ are the observed count, expected count and relative risk of virus *v* and *α*_*v*_ a virus specific intercept term. A multivariate CAR (MCAR) distribution can jointly model *ϕ* by incorporating a between virus covariance matrix Λ^−1^ of dimension *V* × *V* (where *V* is the total number of viruses):

$$\begin{array}{ccc} \phi & ∼ & \text{MVN}(0,{\left[\Omega ⊗\Lambda \right]}^{-1}). \end{array}$$

In this case, Ω = *D* − λ*W*, *ϕ* = {*ϕ*_.1_, …, *ϕ*_.*V*_} and *ϕ*_.*v*_ = {*ϕ*_1*v*_, …, *ϕ*_*Iv*_} [53, 54].

Temporal autocorrelations may be induced in this model, at time point *j*, through the conditional expectation of *ϕ*_*j*_|*ϕ*_*j*−1_

$$\begin{array}{ccc} \phi_{j}|\phi_{j-1} & ∼ & \text{MVN}(s\phi_{j-1},{\left[\Omega ⊗\Lambda \right]}^{-1}). \end{array}$$

The parameter *s* controls the level of temporal autocorrelation such that *s* = 0 implies no autocorrelation whereas *s* = 1 is equivalent to a first order random walk [32]. Typically, where temporal autocorrelations are modelled through the conditional expectation, spatial autocorrelations are modelled through the precision matrix [32].

### Full model

We model monthly time series count data from multiple viruses simultaneously over a nine year period. We index over monthly time intervals and so monthly autocorrelations are modelled in terms of the precision matrix and yearly autocorrelations are modelled in terms of the conditional expectation in a similar fashion to the multivariate spatial-temporal model detailed above. The observed count of virus *v* in month *m* of year *t*, *Y*_*mtv*_ is modelled in terms of the expected count *E*_*mtv*_ and relative risk *RR*_*mtv*_:

$$\begin{array}{c} \begin{array}{cc} Y_{mtv}|E_{mtv},RR_{mtv} & ∼\text{Poisson}(E_{mtv}RR_{mtv})\\ \log(RR_{mtv}) & =\alpha_{v}+\phi_{mtv} \end{array} \end{array}$$

with *α*_*v*_ an intercept term specific to virus *v* and *ϕ*_.*t*._ = {*ϕ*_.*t*1_, …, *ϕ*_.*tV*_} a vector of random effects modelled conditionally through a MCAR prior

$$\begin{array}{ccc} \phi_{.t.}|\phi_{.t-1.} & ∼ & \text{MVN}(s_{v}\phi_{.t-1.},{\left[\Omega ⊗\Lambda \right]}^{-1}). \end{array}$$

This parameterisation of a MCAR model captures both the seasonal trends of each virus via Ω and long-term temporal trends via *s*_1_, …, *s*_*V*_. The conditional expectation of *ϕ*_.*t*._ depends on the previous year *ϕ*_.*t*−1._, capturing long term temporal trends. By allowing dependencies between neighbouring months, we account for seasonality in viral infection frequencies.

#### MCAR prior specification

The covariance structure of the MCAR distribution used to model random seasonal-temporal effects is the Kronecker product of precision matrices Ω and Λ.

The between-virus precision matrix Λ accounts for dependencies between viral relative risks in terms of monthly trends. Wishart priors can be used for unstructured precision matrices such as Λ [55], however, we employed a modified Cholesky decomposition to estimate covariance matrix Λ^−1^:

$$\begin{array}{ccc} \Lambda^{-1} & = & \Sigma \Gamma \Gamma^{T}\Sigma \end{array}$$

where Σ was a diagonal matrix with elements proportional to viral standard deviations and Γ a lower triangular matrix relating to viral correlations [56]. This parameterisation ensured the positive-definiteness of Λ^−1^, although we note that other parameterisations are available [57].

Matrix Ω captures seasonal trends in terms of monthly dependencies defined through a proximity matrix *W*. We will consider two possible constructions of *W*: neighbourhood structure and autoregressive structure.

#### Neighbourhood structure

Assuming neighbouring months are more similar than distant months, *W* can be defined such that *w*_*ij*_ = 1 if months *i* and *j* are neighbouring months and *w*_*ij*_ = 0 if months *i* and *j* are not neighbouring months. Neighbours were fixed as the previous and subsequent three months. Taking a neighbourhood approach, we set

$$\begin{array}{ccc} \Omega_{neigh} & = & D-\lambda W_{neigh} \end{array}$$

where λ is a smoothing parameter and *D* a 12 × 12 diagonal matrix with $D_{i}=\sum_{j}w_{neigh_{ij}}$. The total number of nearest neighbours of month *i* [53, 58].

#### Autoregressive structure

Under this construction, *W* was defined through an autoregressive process and the corresponding matrix denoted by *W*_*auto*_. We set the *ij*th entry of *W*_*auto*_ (*i* ≠ *j*) to be $\rho^{d_{ij}}$ with *d*_*ij*_ the distance between months *i* and *j* and *ρ* a temporal correlation parameter satisfying *ρ* < 1. We defined distance as the number of months between *i* and *j*.

Taking an autoregressive approach, we set

$$\begin{array}{ccc} \Omega_{auto} & = & D-\lambda W_{auto} \end{array}$$

with *D* a diagonal matrix with $D_{i}=\sum_{j}w_{auto_{ij}}.$ We note that these formulations can easily be extended to other MCAR structures [53, 59].

#### Expected counts

We required expected counts of each virus at each time point in this study. Since individual level data were available, a series of logistic regressions were used to estimate the probability of testing positive for a virus at a given time point. For month of the year *m*, the log odds of virus *v*, logit(*p*_*mv*_), was estimated through fixed effects of age, sex and severity (estimated by hospital or general practice submission) and a yearly random effect. The standardised probability of virus *v* in month *m*, $p_{mv}^{s}$, was estimated as

$$\begin{array}{ccc} {\hat{\text{p}}}_{mv}^{s} & = & \sum_{a,s,l,t}\frac{N_{aslt}{\hat{p}}_{mv_{aslt}}}{N_{mv}}. \end{array}$$

where *N*_*aslt*_ was the number of people of age *a*, sex *s* and infection severity *l* in year *t*; ${\hat{p}}_{mv_{aslt}}$ the estimated probability of a person of age *a*, sex *s* with infection severity *l* in year *t* testing positive for virus *v* in month *m*; and *N*_*mv*_ the number of swabs tested for virus *v* in month *m*. The estimated probabilities of each virus in each month are therefore standardised for age, sex and severity and account for yearly differences in circulation.

The expected count for virus *v* in month *m* of year *t* was then

$$\begin{array}{c} E_{mtv}=N_{mtv}{\hat{\text{p}}}_{mv}^{s} \end{array}$$

with *N*_*mtv*_ the number of of patient episodes of illness tested for virus *v* in month *m* in year *t*.

#### Estimating model parameters

This model was implemented in jags [60] using the R2jags package [61] in R [62]. All results are averaged across five independent chains. In each chain, we took 50,000 thinned draws across 500,000 iterations after a burn-in period of 300,000 iterations. R code used to fit models is provided (S1 Appendix). We note that the multivariate intrinsic Gaussian CAR prior distribution is fully specified in GeoBUGS [63]. However, our approach allows for other parameterisations of the MCAR distribution providing more flexibility in separating monthly and yearly temporal dependencies.

#### Multiple comparison correction

For each covariance parameter, higher posterior density intervals (HPDI) were estimated. Posterior probabilities were then estimated to assess the probability of zero being included in each interval, synonymous to Bayesian p-values defined in terms of lower tail posterior probabilities [64, 65]. Covariance parameters with a posterior probability less than 0.05 were deemed different from zero [64]. In order to control for multiple comparisons, covariance parameters with an adjusted probability, controlling the false discovery rate [64, 66], less than 0.05 were deemed different from zero and used as support for a significant covariance between the corresponding viruses.

### Simulation study

The specific aim of this paper was to estimate the between-virus covariance matrix Λ^−1^. We prove the validity of our proposed model (Fig 1) in modelling multivariate time series data through simulating data from three viral infections ranging from independence to moderately high correlations. We illustrate that this method had power to detect dependent time series data whilst controlling the Type 1 error rate.

We began by simulating individual level data reflecting the virological diagnostic data. For each sample, an age, sex and severity were drawn from the observed virological diagnostic data distributions [38]. Regression coefficients used to estimate the probability of each virus were drawn such that *β*_*intercept*_ = 0, *β*_*age*_ ∼ *N*(0, 0.1), *β*_*gender*_ ∼ *N*(0, 0.1) and *β*_*severity*_ ∼ *N*(0, 0.1). Within each year, we randomly sampled between 20 and 200 samples per month per virus in order to reflect differing testing frequencies within and between viruses. Standardised probabilities of each virus within each month ${\hat{p}}_{mv}^{s}$ were then estimated using the methods described in the Expected counts section. Expected counts were taken as the product of the standardised probabilities and the number of samples taken within that month for the corresponding virus.

Monthly effect sizes were simulated using the sarima package [67] in R [62]. We choose this package due to its flexibility in simulating seasonal non-stationary time series data. We were able to combine differencing (or order *d*) with an autoregression (of order *p*) and a moving average model (of order *q*) to obtain a non-seasonal ARIMA model. In addition seasonal components were included through seasonal differencing (*D*), autoregression (*P*) and a moving average model (*Q*) over period *m* therefore simulating from a SARIMA(*p*, *d*, *q*)(*P*, *D*, *Q*)_12_ with period 12 since we are dealing with monthly data. Within each simulation, we used differencing *d* = 1 with a second or first order autoregression and moving average *p*, *q* ∈ {1, 2}. Likewise, we used either no or a seasonal differencing *D* ∈ {0, 1} and no or a first order autoregression and moving average *p*, *q* ∈ {0, 1}. These parameter settings allowed for a wide range of seasonal and non-stationary time series data. Fig 2 provides examples of simulated time series data under these parameter settings.

Random effects *ϕ* were drawn from multivariate normal distributions with yearly smoothing parameters and monthly smoothing parameter *s*_1_, *s*_2_, *s*_3_ and λ simulated uniformly between 0 and 0.9 and precision matrix equal to the Kronecker product of matrices Ω and Λ. Matrix Ω was dependent on the choice of structure used to simulate data. In this case we simulated from both the neighbourhood and autoregressive structure (Fig 1). In the case of the autoregressive structure, we simulated *ρ* uniformly between 0 and 0.9 (method described in MCAR prior specification section).

Matrix Λ was the virus correlation matrix that we aimed to estimate. We simulated data from three viruses with one virus pair, virus 1 and virus 2, non-independent of each other but both independent of the remaining virus, virus 3. We explored a variety of correlations between virus 1 and virus 2 ranging from 0.2 to 0.5. This range was chosen to reflect weakly related viruses (0.2) to moderate to strongly related viruses (0.5). We anticipated that as the strength of correlation increased, the power would also increase whilst still controlling the type 1 error rate.

Relative risks were then taken additively as the exponential of virus intercept terms *α*_1_, *α*_2_, *α*_2_ simulated uniformly and random effects *ϕ*. Observed counts were the product of expected counts and relative risks. Fig 3 illustrates observed and expected counts from three viruses.

We fitted both models (Fig 1, neighbourhood and autoregressive structure) to data simulated through both structures with or without a multiple comparison correction creating eight possible simulation and estimation scenarios (Table 1). In each case we simulated and estimated 100 times. Each model was fitted in jags [60] using the R2jags package [61] in R [62] (S1 Appendix). All results are averaged across two independent chains. In each chain, we took 3000 thinned draws across 300,000 iterations after a burn-in period of 200,000 iterations.

Under each scenario we estimated higher posterior density intervals (HPDI) for covariance parameters (${\hat{\Lambda }}_{12}$,${\hat{\Lambda }}_{13}$ and ${\hat{\Lambda }}_{23}$). Posterior probabilities were then estimated to assess the probability of zero being included in each interval, synonymous to Bayesian p-values defined in terms of lower tail posterior probabilities [64, 65]. Covariance parameters with a posterior probability less than 0.05 were deemed different from zero [64]. In order to control for multiple comparisons, covariance parameters with an adjusted probability, controlling the false discovery rate [64, 66], less than 0.05 were deemed different from zero and used as support for a significant covariance between the corresponding viruses.

## Supporting information

S1 Appendix R code used to fit neighbourhood and autoregressive models. R code used to fit models described in Fig 1. Models were written and fitted in jags.
(R) Click here for additional data file.

S2 Appendix Observed values plotted agained fitted values. Fitted values based on the best fitting autoregressive model plotted against observed values with the line of equality (*y* = *x*). Correlations and p-values between fitted and observed values are given for each virus.
(PDF)
