# Supplementary figures and images for: Estimation of temporal covariances in pathogen dynamics using Bayesian multivariate autoregressive models
Source: PLoS Comput Biol. 2019 Dec 13;15(12):e1007492. doi: 10.1371/journal.pcbi.1007492 (PMC6934324; doi:10.1371/journal.pcbi.1007492)

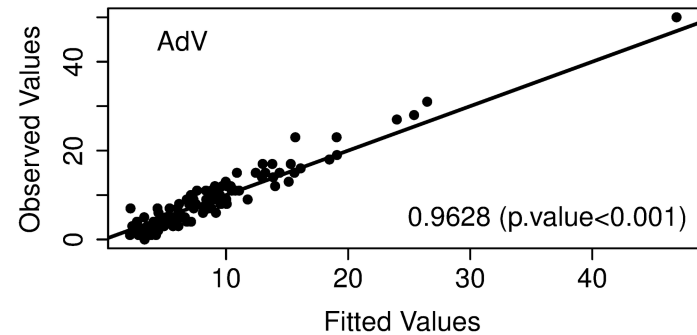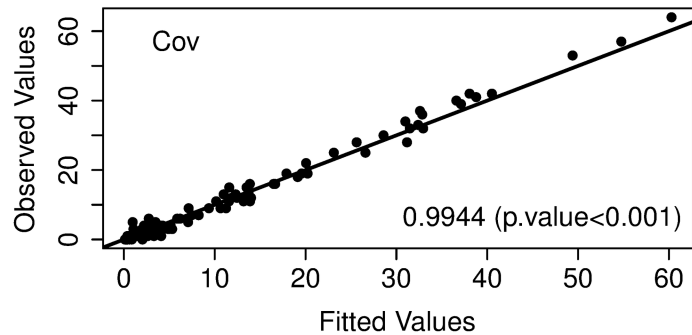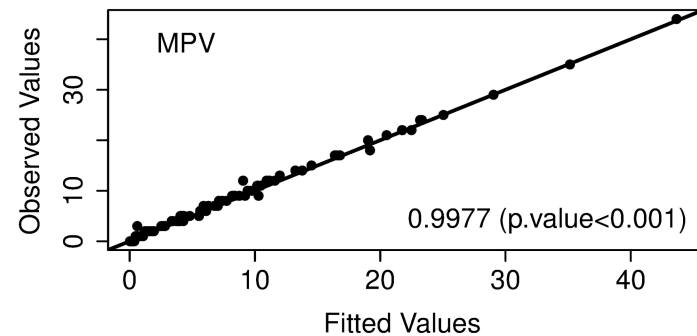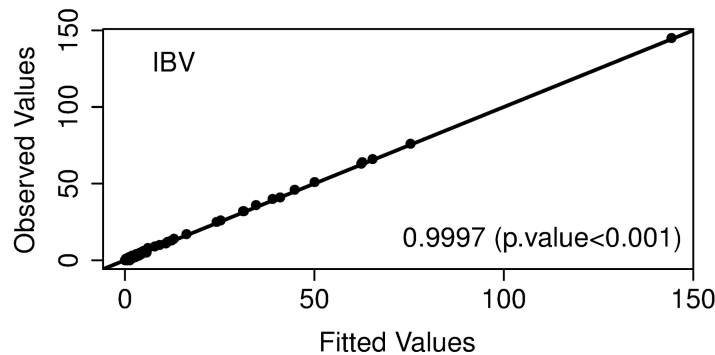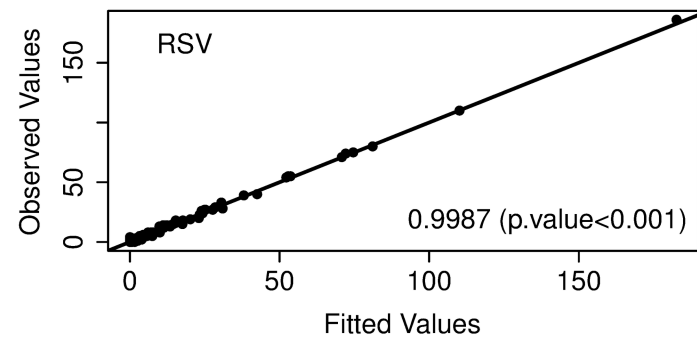

Supplement: S2 Appendix — Fitted values based on the best fitting autoregressive model plotted against observed values with the line of equality (y = x). Correlations and p-values between fitted and observed values are given for each virus. (PDF) [file pcbi.1007492.s002.pdf]
